# Application of a framework to guide genetic testing communication across clinical indications

**DOI:** 10.1186/s13073-021-00887-x

**Published:** 2021-04-29

**Authors:** Miranda L. G. Hallquist, Eric P. Tricou, Kelly E. Ormond, Juliann M. Savatt, Curtis R. Coughlin, W. Andrew Faucett, Laura Hercher, Howard P. Levy, Julianne M. O’Daniel, Holly L. Peay, Melissa Stosic, Maureen Smith, Wendy R. Uhlmann, Hannah Wand, Karen E. Wain, Adam H. Buchanan

**Affiliations:** 1 Geisinger, 100 N Academy Blvd, Danville, PA 17822 USA; 2 Department of Genetics and Stanford Center for Biomedical Ethics, Center for Academic Medicine, Stanford University School of Medicine, 453 Quarry Road, Stanford, CA 94304 USA; 3 University of Colorado Department of Pediatrics and Center for Bioethics and Humanities, University of Colorado Anschutz Medical Campus, Aurora, Colorado 80045 USA; 4 Sarah Lawrence College Joan H. Marks Graduate Program in Human Genetics, 1 Mead Way, Bronxville, NY 10708 USA; 5 Johns Hopkins University Division of General Internal Medicine and McKusick-Nathans Institute of Genetic Medicine, 0753 Falls Rd, Suite 325, Lutherville, MD USA; 6 Department of Genetics Genetic Medicine Building, University of North Carolina at Chapel Hill, 120 Mason Farm Rd, CB # 7264, Chapel Hill, NC 27514 USA; 7 RTI International, 3040 E Cornwallis Rd, Research Triangle Park, NC 27709 USA; 8 DotLab, 780 E Main St, Suite 1, Branford, CT 06405 USA; 9 Northwestern University Feinberg School of Medicine, 310 E. Superior St., Chicago, IL 60611-3008 USA; 10 Department of Internal Medicine, Division of Genetic Medicine, University of Michigan Medicine, 300 North Ingalls, NI3 A03, SPC 5419, Ann Arbor, MI 48109-5419 USA

**Keywords:** Genetic testing, Genetic counseling, Informed consent, Results disclosure, Access, Service delivery

## Abstract

**Background:**

Genetic information is increasingly relevant across healthcare. Traditional genetic counseling (GC) may limit access to genetic information and may be more information and support than some individuals need. We report on the application and clinical implications of a framework to consistently integrate genetics expertise where it is most useful to patients.

**Methods:**

The Clinical Genome Resource’s (ClinGen) Consent and Disclosure Recommendations (CADRe) workgroup designed rubrics to guide pre- and post-genetic test communication. Using a standard set of testing indications, pre- and post-test rubrics were applied to 40 genetic conditions or testing modalities with diverse features, including variability in levels of penetrance, clinical actionability, and evidence supporting a gene-disease relationship. Final communication recommendations were reached by group consensus.

**Results:**

Communication recommendations were determined for 478 unique condition-indication or testing-indication pairs. For half of the conditions and indications (238/478), targeted discussions (moderate communication depth) were the recommended starting communication level for pre- and post-test conversations. Traditional GC was recommended pre-test for adult-onset neurodegenerative conditions for individuals with no personal history and post-test for most conditions when genetic testing revealed a molecular diagnosis as these situations are likely higher in complexity and uncertainty. A brief communication approach was recommended for more straightforward conditions and indications (e.g., familial hypercholesterolemia; familial variant testing).

**Conclusions:**

The CADRe recommendations provide guidance for clinicians in determining the depth of pre- and post-test communication, strategically aligning the anticipated needs of patients with the starting communication approach. Shorter targeted discussions or brief communications are suggested for many tests and indications. Longer traditional GC consultations would be reserved for patients with more complex and uncertain situations where detailed information, education, and psychological support can be most beneficial. Future studies of the CADRe communication framework will be essential for determining if CADRe-informed care supports quality patient experience while improving access to genetic information across healthcare.

**Supplementary Information:**

The online version contains supplementary material available at 10.1186/s13073-021-00887-x.

## Background

To achieve informed decision-making about genetic testing and support patients in integrating genetic results into their health care, the traditional genetic counseling (GC) model includes providing information pre-test about the condition being tested, limitations and risks of the testing, possible test results and their significance for medical management, and potential implications for family members [1–3]. Traditional GC also includes disclosing the result post-test and discussing the interpretation in the context of the individual’s personal and family history, reviewing the implications for medical management, explaining the significance for family members, assessing understanding, providing psychological support, clarifying testing limitations, and providing referrals to additional health care providers as appropriate [3–5]. The provision of GC has been associated with improved medical and psychological outcomes, such as increased adherence to risk management, enhanced family communication, and decreased worry and anxiety, and has been supported by prominent professional organizations [6–11].

There is a growing body of evidence that patients may also benefit from alternative approaches to genetic test discussions, suggesting that some patients or situations may not require traditional GC. Studies evaluating genetic testing care models to facilitate treatment decisions for individuals diagnosed with epithelial ovarian cancer have found significant increases in the proportion of patients who completed genetic testing, faster turnaround times for test results, and continued patient satisfaction with streamlined pre-test education and immediate testing with an oncologist rather than the traditional referral method of a separate pre-test GC appointment [12, 13]. Furthermore, alternative service delivery models that shorten clinician-provided GC, such as the addition of pre-test education aids like videos [14] and chatbots [15], and results discussions via web-based platforms rather than in-person [16] have shown similar outcomes to traditional GC.

Shifting consent and disclosure approaches largely focus on how genetics professionals conduct individual consults without taking a broader view to strategically determine the clinical situations in which patients may benefit most from genetics provider expertise. As genomics information is increasingly incorporated into health care across multiple specialties, the traditional GC approach is likely unsustainable as it continues to be time-intensive, with a majority of pre-test GC appointments lasting over 45 min [17] and concerns for workforce shortages and access issues existing in some areas of the country for both GC [18] and MD geneticist consultations [11, 19, 20]. A framework that guides the application of genetics expertise where it is most useful to patients could balance multiple goals, including increasing access to genetic information, providing quality patient experience for individuals pursuing genetic testing, and improving clinical outcomes when incorporating genetic results into their care.

The Consent and Disclosure Recommendations working group (hereafter “CADRe”) is part of the Clinical Genome Resource (ClinGen), a National Institutes of Health-funded consortium of clinicians, laboratorians, and researchers that is building an open-access resource for defining the relevance, actionability, and communication of genomic variants (clinicalgenome.org) [21]. CADRe has developed a framework designed to provide guidance regarding the genetic conditions and clinical indications in which patients would benefit most from traditional GC where detailed discussion, complex test selection, and psychological support are provided, and, conversely, the conditions and testing indications where a more abbreviated discussion may be appropriate. Recognizing that each clinician-patient interaction is unique, the CADRe recommendation is intended as a starting point; the ultimate communication approach should be tailored to the needs and preferences of individual patients and clinicians. For example, if a clinician is concerned about the potential for a negative psychological response related to a patient’s personal history, a referral to traditional GC either pre- or post-test might be warranted even when the communication approach recommended by the CADRe framework is a targeted discussion or brief communication [22].

**Fig. 1:**
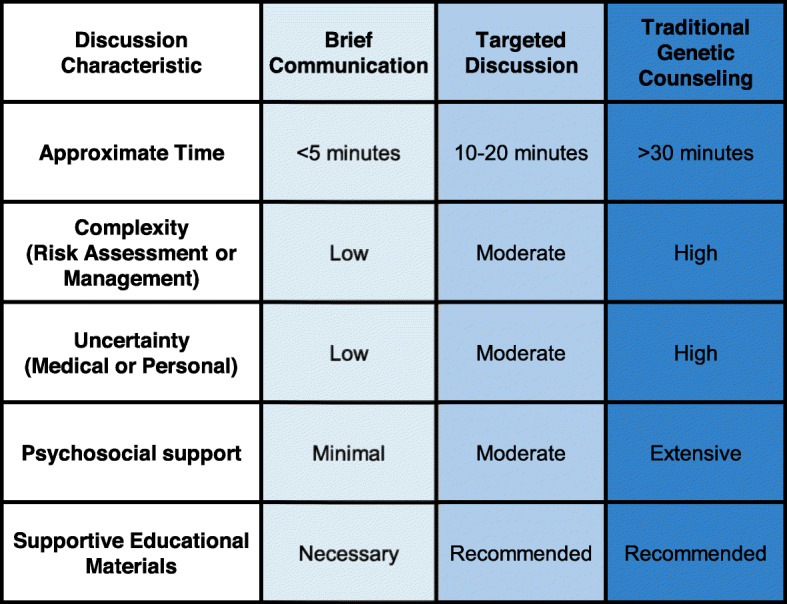
CADRe communication levels

CADRe operationalized the framework as two rubrics (pre- and post-test) that were then formatively evaluated with patient and provider stakeholders [23]. The rubrics consider features such as management complexity, medical actionability, and psychological impact for a given testing situation to determine one of three communication approaches at pre- and post-testing: (1) traditional GC, (2) targeted discussion, or (3) brief communication (Fig. 1). While traditional GC would be provided by a clinician with genetics expertise, the targeted discussion and brief communication conversations could be completed by any clinicians (with or without formal genetics training) who are knowledgeable and experienced with the content. The framework supports multiple modalities for communication at any level, such as in-person, telephone, video, and web portal. The post-test rubric is specific to results that are classified as either (1) pathogenic/likely pathogenic and are indicative of a molecular diagnosis and/or risk (hereafter “P/LP”) or (2) benign/likely benign/no variant detected (“B/LB”) in genes with established disease relationships. Results that are classified as variants of uncertain significance, either due to limited variant information or limited/unknown gene-disease relationships, require clinician assessment on a case-by-case basis to determine the best pathway for discussion of results and follow-up based on the overall clinical situation, patient context, and specific variant identified.

Here, we report the communication approaches recommended using the CADRe framework to classify a variety of genes, genetic conditions, testing indications, and testing modalities, and discuss the potential implications on clinical care.

## Methods

### CADRe

The 18-person CADRe workgroup includes genetic counselors, physician geneticists, and bioethicists with specialized genetics training. CADRe co-chairs invited participation in the workgroup through their professional networks based on invitees’ research or clinical expertise. Membership spans many work settings (academic medical centers, community health systems, and industry) and genetic specialties (general medical genetics, prenatal, women’s health, adult genetics, oncology, cardiogenetics, and neurogenetics).

### The rubrics

**Fig. 2:**
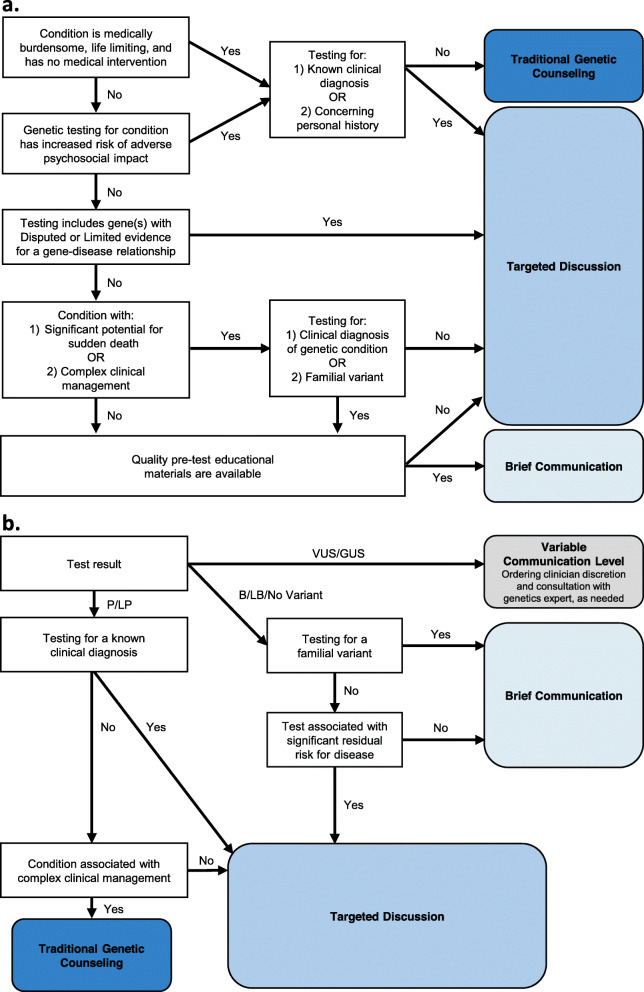
CADRe communication rubrics. **a** Pre-test discussion rubric. **b** Post-test discussion rubric. P/LP, pathogenic/likely pathogenic results that are indicative of a molecular diagnosis and/or risk; B/LB, benign/likely benign; VUS/GUS, variant of uncertain significance/gene of uncertain significance

The pre- and post-test rubrics previously described [23] were iteratively updated with additional considerations that CADRe believed would influence the recommended communication approach (Fig. 2). The initial CADRe pre-test rubric included the following considerations of a condition: (1) if it is medically burdensome, is life limiting, and has no medical intervention; (2) if genetic testing for the condition has been documented to increase the risk of adverse psychosocial impact; (3) if a condition has significant potential for sudden death or complex clinical management; and (4) if there are quality pre-test educational materials available. As CADRe applied the pre-test rubric in the current study, two additional considerations were added: if the testing modality includes any gene(s) with disputed or limited evidence for a gene-disease relationship, and the indication for which the individual was pursing testing (e.g., an individual having testing due to a personal diagnosis or due to a known familial variant). The initial CADRe post-test rubric included the following considerations: (1) if the result was P/LP, B/LB, or uncertain; (2) if the test was associated with a significant residual risk for disease; and (3) if the condition was associated with complex clinical management. Applying the post-test rubric in the current study led to the additional consideration of testing indications of a known clinical diagnosis or known familial variant. Throughout the classification process, CADRe assumed that clinicians coordinating genetic testing are proficient in identifying a genetic risk, choosing an appropriate genetic test, communicating the test result, and assessing patient understanding and comfort level with the genetic testing.

### Applying the rubrics

#### Selecting the genes/conditions

The CADRe rubrics were applied to several categories of conditions/genes (Table 1). Each category was chosen to highlight different potential considerations, including variable levels of penetrance, evidence supporting a gene-disease relationship [29], and clinical actionability (the recognition of interventions which may prevent, delay onset of, or reduce burden of disease [30]).

**Table 1:** CADRe rubric application

Category	Genes included	Rationale
Genes included in the American College of Medical Genetics secondary findings (ACMG SF) v2.0 [24]	Cancer risk: *BRCA1*, *BRCA2*, *MLH1*, *MSH2*, *MSH6*, *PMS2*, *RET*, *BMPR1A*, *SMAD4*, *TP53*, *MEN1*, *TSC1*, *TSC2*, *PTEN*, *STK11*, *APC*, *VHL*, *NF2*, *RB1*, *SDHD*, *SDHAF2*, *SDHC*, *SDHB*, *MUTYH*, *WT1* Cardiovascular risk: *FBN1*, *TGFBR1*, *TGFBR2*, *SMAD3*, *ACTA2*, *MYH11*, *COL3A1*, *LDLR*, *APOB*, *PCSK9*, *RYR2*, *KCNQ1*, *KCNH2*, *SCN5A*, *MYBPC3*, *MYH7*, *TNNT2*, *TNNI3*, *TPM1*, *MYL3*, *ACTC1*, *PRKAG2*, *MYL2*, *LMNA*, *PKP2*, *DSP*, *DSC2*, *TMEM43*, *DSG2* Metabolic: *GLA*, *OTC*, *ATP7B* Other: *RYR1*, *CACNA1S*	Well described, evidence for medical intervention, commonly evaluated [25]
Moderate penetrance cancer risk	Breast cancer risk: *ATM*, *CHEK2*, *PALB2, BARD1* Colon cancer risk: *GREM1*, *POLD1*, *POLE*	Commonly included on genetic testing panels with similar phenotypes as some genes in the ACMG SF v2.0 [24, 26]
Moderate evidence cardiomyopathy risk [27]	Cardiovascular risk: *CSRP3*, *TNNC1*, *JPH2*	Commonly included on genetic testing panels with similar phenotypes as some genes in the ACMG SF v2.0 [24, 26]
Genes that have limited evidence of gene-disease association for hypertrophic cardiomyopathy [27]	Limited evidence hypertrophic cardiomyopathy risk: *TTN*, *KLF10*, *MYPN*, *ANKRD1*	Included as preliminary evidence add-on genes on genetic testing panels [26]
Genes that have refuted or disputed evidence of gene-disease association for breast cancer risk [28]	*BRIP1*, *RAD51C*	Included on broad cancer panels, may not relate to indication for testing, and yet would have management recommendations if a pathogenic/likely pathogenic variant is identified [26]
Genes associated with risk for neurodegenerative disorders	*HTT*, *APP*, *PSEN1*, *PSEN2*, *MAPT*, *GRN*, *SOD1*, *c9orf72*, *ApoE*	Not clinically actionable
Exome	Not Applicable	Increasingly common genetic testing in which multiple genetic conditions are under consideration

#### Process for evaluation

Based on our earlier work that suggested that many of the variables in the CADRe rubric could be influenced by the indication for genetic testing [23], four standard indications for genetic testing were examined: (1) individual with a clinical diagnosis of a genetic condition where genetic testing was expected to be confirmatory (hereafter “confirmatory testing”), (2) individual with a personal history suggestive of a genetic condition (“suggestive personal history”), (3) unaffected individual with a suggestive family history in which the proband was not known to have undergone genetic testing (“suggestive family history”), and (4) unaffected individual with a known P/LP variant in a family member (e.g., predictive or cascade testing, “familial variant”). Condition-specific indications were added or removed as applicable (e.g., tumor screening positive for Lynch syndrome risk; confirmatory testing only used for conditions with clinical diagnostic criteria). All CADRe recommendations were thus formulated specific to a condition-indication pair or testing modality-indication pair (e.g., familial hypercholesterolemia-confirmatory testing; exome testing-suggestive personal history). For the indication of confirmatory testing, CADRe considered the issues surrounding consent and disclosure *as related to the genetic test* and did not consider issues related to living with, and adapting to, the condition or medical management recommendations since these would have been discussed with the patient at the time of receiving the clinical diagnosis, and genetic test results would not likely change the patient’s clinical diagnosis or current management plan. This is in contrast to the other three indications in which identification of a P/LP variant would be a new molecular diagnosis of a hereditary condition or risk and, therefore, likely to change a patient’s medical management.

The CADRe rubric was operationalized as a set of questions using the online survey tool SurveyMonkey (SurveyMonkey, San Mateo, CA) to provide the rubric-recommended communication level (Additional file 1: Supplementary Methods). Workgroup members were asked to agree or disagree with the preliminary level of communication provided by the rubric; if they disagreed, they were prompted to provide an alternative communication approach and reason for recommending that approach. Five-member subgroups of CADRe were formed for completion of the rubrics. For classification of the American College of Medical Genetics secondary findings (ACMG SF) v2.0 conditions [24], subgroups comprised multiple specialties. For classification of genes with moderate penetrance, varying levels of evidence of gene-disease association (moderate, limited, refuted or disputed), neurodegenerative risk, and exome sequencing, subgroups of members with clinical expertise in those areas were formed. For each condition or test, subgroup members typically completed 12 classifications: pre-test rubric for each of the four standard indications, post-test with P/LP results rubric for each of the indications, and a post-test B/LB rubric for each of the indications. Since variants of uncertain significance are recommended to be assessed by clinicians on a case-by-case basis, no rubrics were completed for these types of results.

After individual subgroup members completed the online rubrics, results were collated and reviewed within the subgroup. Subgroups developed a consensus recommendation through discussion of the collated rubric results, requiring agreement of at least three of the five subgroup members. This method of developing consensus through discussion after initial assessment by individual experts is similar to methods described by the ClinGen Actionability Working Group [30]. The subgroup recommendation was then presented to the full CADRe working group membership for final approval. If a subgroup was unable to reach consensus, the discussion was brought to the full CADRe membership for resolution. When subgroups were unable to reach consensus, the discussion typically centered around how to apply the CADRe rubric to a given testing scenario. Often, these discussions provided an opportunity to update the rubric and the subsequent CADRe framework. For example, when considering the post-test communication recommendation for someone with a personal history suggestive of Long QT syndrome with no significant variant identified on genetic testing, the subgroup conversation focused on whether the correct testing had been ordered. If so, then the subgroup thought that targeted discussion would be appropriate as the rubric suggested. If incomplete testing had been done, however, some members argued that traditional GC would be most appropriate for addressing the consideration of additional genetic testing. This conversation was the catalyst for CADRe to adopt the overarching assumption that clinicians coordinating genetic testing are proficient in identifying a genetic risk and choosing the appropriate genetic test.

Two authors (MH and ET) reviewed all communication recommendations to ensure consistent application of the rubric between subgroups. When recommendations differed from the overall CADRe trends, they were re-assessed by the full CADRe membership in a process similar to the initial classification workflow.

## Results

**Fig. 3:**
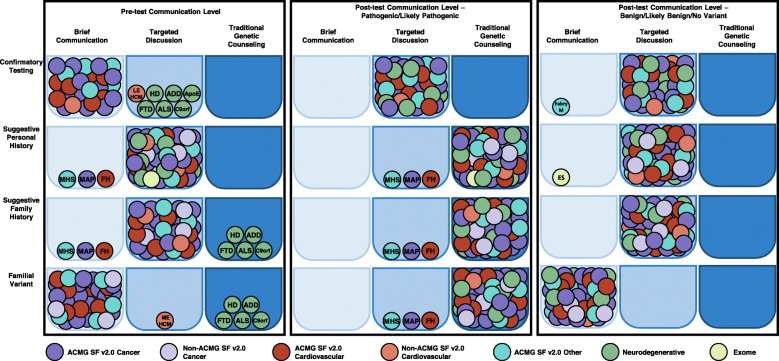
CADRe pre- and post-test rubric classifications by indication. Each bin represents the classifications for a communication recommendation (horizontal axis) and indication (vertical axis) and contains the circles of individual curations for a gene, condition, or testing modality. Standard indications classified were as follows: (1) confirmatory testing: individual with a clinical diagnosis of a genetic condition where genetic testing was expected to be confirmatory; (2) suggestive personal history: individual with a personal history suggestive of a genetic condition; (3) suggestive family history: unaffected individual with a suggestive family history in which the proband was not known to have undergone genetic testing; and (4) familial variant: unaffected individual with a known P/LP variant in a family member (e.g., predictive or cascade testing). Condition-specific indications were added or removed as applicable. ADD, autosomal dominant dementia; ALS, amyotrophic lateral sclerosis; ApoE, ApoE genotyping; c9orf, c9orf mediated FTD/ALS; ES, exome sequencing; Fabry M, Fabry testing in individual with male sex due to a chromosomal complement of XY, XYY, or other that causes risk for Fabry; FH, familial hypercholesterolemia; FTD, frontotemporal dementia; HD, Huntington Disease; LE HCM, limited evidence hypertrophic cardiomyopathy; MAP, *MUTYH*-associated polyposis; MHS, malignant hyperthermia susceptibility; ME HCM, moderate evidence hypertrophic cardiomyopathy

Figure 3 provides a visual summary of results. A total of 40 sets of rubrics were completed online by workgroup members. Each set contained a rubric for pre-test communication, post-test communication about P/LP results, and post-test communication about results in which no clinically significant variants are reported (three panels in Fig. 3). An individual set of rubrics was specific for a gene (e.g., *RB1*), condition (e.g., *MLH1*, *MSH6*, *MSH2*, *PMS2*, and *EPCAM* were classified together as Lynch syndrome), or testing modality (e.g., exome) and is represented by a circle in Fig. 3. A set of four common testing indications was included for most rubrics, with indications added or removed as appropriate for the condition or testing modality. Each bin in Fig. 3 represents the classifications for a communication recommendation (horizontal axis) and indication (vertical axis) and contains circles representing a gene, condition, or testing modality. For example, in the panel with the pre-test communication levels, a circle representing Lynch syndrome can be found in the bins for brief communication-confirmatory testing, targeted discussion-suggestive personal history, targeted discussion-suggestive family history, and brief communication-familial variant testing. Additional results are summarized in Additional file 2: Table S1 and Additional file 3: Table S2.

Overall, CADRe completed classification of 478 condition-indication or testing-indication pairs (120 traditional GC, 238 targeted discussion, 116 brief communication). Of these classifications, 22 (4.6%) differed from the overall CADRe trends and were re-assessed by the working group. Four of the 22 classifications were unchanged after re-assessment, four were updated with a decreased level of communication (from targeted discussion to brief communication), and 14 were updated with an increased level of communication (three from brief communication to targeted discussion, and 11 from targeted discussion to traditional GC).

### Pre-test (consent) recommendations

Traditional GC was the recommended pre-test communication approach for adult-onset, clinically non-actionable, neurodegenerative conditions when an individual is unaffected and has a suggestive family history or a known familial variant. This was due to the perceived complexity for individuals as they make decisions about pursuing predictive testing. While data was limited for many of the neurodegenerative conditions we examined, there is clear evidence suggesting increased psychological risks in predictive testing for at least some of the genes reviewed. For example, individuals with Huntington disease risk (*HTT*) have been shown to have increased risks for both short- and long-term anxiety, depression, and even suicide [31]. In contrast, for individuals who already have a known clinical diagnosis or a suggestive personal history of a neurodegenerative condition, a targeted discussion pre-test communication approach was recommended. The targeted discussion recommendation assumes that the diagnosis, prognostic information, and some psychological issues related to the diagnosis have likely been raised, thus making the conversation less complex than a discussion about predictive testing.

Targeted discussions were the recommended pre-test communication approach for genetic testing of nearly all of the medically actionable conditions with moderate to high penetrance and established gene-disease relationships when the indication was suggestive personal or family history. Targeted discussion was also recommended for tests that include genes with limited or unknown gene-disease relationships (e.g., exome sequencing or large panel testing). This was generally because these situations have moderate levels of medical uncertainty and/or complexity. For example, pre-test discussions for exome sequencing include the complexity of broad genomic testing, the chance of secondary findings, and uncertainty in results interpretation such as identification of variants of uncertain significance or variants in genes with unclear disease association.

Three of the conditions reviewed—familial hypercholesterolemia (FH), *MUTYH*-associated polyposis (MAP), and malignant hyperthermia susceptibility (MHS)—were notable exceptions where a brief communication approach was recommended for pre-test communication. These conditions were identified to have lower clinical complexity given well-established treatment or prevention. FH was considered less complex as there is primarily a single organ system impacted (cardiovascular) and the treatment is well understood (lipid lowering therapy) [32]. Likewise, MAP primarily impacts colon polyp development, a single organ system, and colonoscopy is well-established and efficacious surveillance [33]. While a malignant hyperthermia event is systemic and impacts multiple organs, the prevention of that event (avoidance of triggering anesthetic agents) is well established and straightforward [34].

Finally, the CADRe framework suggests a brief communication approach pre-test for confirmatory genetic testing, and for familial variant testing for actionable conditions with moderate to high penetrance and an established gene-disease relationship. Individuals seeking testing for these indications are likely to be familiar with the diagnosis and management of the condition through lived experience (their own or a family members’), which could make many pre-test discussions low in both complexity and uncertainty.

### Post-test (results disclosure) recommendations

To communicate P/LP genetic testing results, the CADRe rubric most often recommended a brief disclosure by the ordering clinician, followed by traditional GC. This recommendation was made for all indications except confirmatory testing for a known clinical diagnosis, and all conditions except those identified as less clinically complex (FH, MAP, and MHS). Traditional GC was seen as important for discussing the majority of P/LP results to provide patients with detailed information about natural history, penetrance, medical surveillance and treatment, and implications for family members, and to provide psychological support as they are coping with learning about a new molecular diagnosis and its impact on their family. If genetic testing had been performed in relation to a known clinical diagnosis, however, targeted discussion was suggested to follow up a P/LP result. These situations were considered less uncertain since the genetic testing result aligns with what was expected based on clinical findings and would be unlikely to change the medical management previously discussed based on the known clinical diagnosis. In these situations, the main area of complexity is discussing the implications of the genetic test result for family members.

When disclosing B/LB results to patients with suggestive personal or family history and for those who had confirmatory testing, a targeted discussion was recommended. It is important to explain the uncertainty related to limitations of the testing, consider additional genetic testing, and discuss medical management related to the residual risk for disease. There were three scenarios that did not follow this trend, where a brief communication was recommended for post-test results discussion: (1) an exome result indicates no clinically significant variant, (2) familial variant testing does not identify the known variant (a “true negative”), and (3) genetic testing for Fabry disease shows no clinically significant variant results for a male with a diagnosis of Fabry based on enzyme testing. (Here, we mean an individual of male sex due to a chromosomal complement of XY, XYY, or other that causes risk for Fabry, an X-linked condition). CADRe recommended a brief communication in these three situations since the management of the individual would continue based on their personal history regardless of the negative genetic test results. Thus, these particular situations are lower in complexity and uncertainty.

## Discussion

Integrating genetics across healthcare in a way that provides quality patient experience, expands access, and improves clinical outcomes calls for a framework to apply genomics expertise where it is likely to be most beneficial to patients and clinicians. To provide such guidance for communication related to genetic testing, CADRe applied a set of rubrics to genetic conditions with a variety of features, including variability in actionability, penetrance, gene-disease relationship, and testing modality. We anticipated that the communication approach would be related to characteristics of specific genes and conditions. However, we discovered that the gene and condition characteristics were less impactful than the *indication for testing*, which was the main predictor of the recommended communication approach across conditions, penetrance, gene-disease evidence levels, and types of testing. The indication for testing strongly influenced the CADRe communication recommendation because indication is related to the uncertainty and complexity that a patient may be facing in a given situation. For most conditions and indications, targeted discussions were the recommended starting communication level for both pre- and post-test conversations. Traditional GC was recommended for indications that present more complex and uncertain situations such as predictive testing for a neurodegenerative condition or results that reveal a new molecular diagnosis of a genetic condition. Brief communication was recommended for more straightforward pre- and post-test indications or modalities such as familial variant testing or B/LB exome sequencing results.

Uncertainty is a composite concept that incorporates multiple levels of experience relating to having imperfect or unknown information [35]. Uncertainty may arise in genetic testing from many sources, including multiple causes of a particular phenotype (locus and allelic heterogeneity, environmental influences), variable effects or a particular genotype (pleiotropy, penetrance, variable expressivity, anticipation), and the scientific uncertainty of diagnostic and prognostic information related to gene-disease association or variant pathogenicity [36]. Situations with increased uncertainty can lead to more complex decision making (pre-test) and challenges with comprehension of results, coping, and adaptation (post-test) and have wide-ranging implications for how individuals understand and adapt to genetic information and how they make meaning out of genetic risk for themselves and their family members. These complex genetic testing situations are those that benefit from the specialized training of genetics providers to assist patients with making decisions about genetic testing and support patients in learning significant genetic findings and subsequently acting on those results [19]. For example, the important outcomes of patients *understanding* and *applying information* to (1) *make decisions,* (2) *manage a condition*, and (3) *adapt to their situation* are highlighted in the Reciprocal Engagement Model of the GC process [37].

The CADRe approach to triaging the communication about genetic information aligns the anticipated needs of patients with more or less detailed communication from clinicians. By changing the amount and depth of both content and emotional support between our proposed communication approaches, CADRe suggests a shift in the way clinicians communicate about most genetic testing. Providing traditional GC support to individuals who are in the most uncertain and complex testing situations allows genetics clinicians the opportunity to prioritize the informational and emotional support needs of patients in these circumstances. Triaging situations with lower uncertainty and complexity to more abbreviated targeted discussions or brief communications will likely still provide an appropriate level of care for these patients in a less time-intensive manner. Viewing communication needs through the lens of uncertainty and complexity as experienced by patients, their families, and their clinicians mirrors the broader practice of medicine, where specialists are relied upon to provide care for complex management in their areas of expertise [38].

In addition to the potential to improve patient experience with genetic testing, using a model in which targeted discussions are the starting point for conversations about genetic testing for most conditions and testing indications has significant implications for genetic testing service delivery. Since targeted discussions provide a moderate level of depth and psychological support, it is likely the length of a “targeted discussion” appointment would be substantially shorter than those currently occurring in a more traditional genetic counseling approach. This could both increase capacity for genetics provider clinic volume by reducing the amount of time spent with a large proportion of patients and could be implemented by clinicians who do not have genetic-specific training but who are comfortable with the coordination of genetic testing as part of their practice. For example, an oncologist may be comfortable with ordering a panel test for individuals with a diagnosis of ovarian cancer, or a cardiologist may order panel testing for patients clinically diagnosed with hypertrophic cardiomyopathy. The combination of increasing capacity of genetics clinics and non-genetics providers ordering testing within their clinical area would potentially increase patient access to genetic testing information at the point-of-care and address clinical bottlenecks due to workforce shortage issues for genetics-trained providers.

Implementation of the targeted discussion and brief communication approaches will require additional support, infrastructure, and continued development of educational materials for clinicians who order genetic testing. To begin to develop that support for targeted discussions, it is important to have consensus on the necessary pieces of informed consent for genetic testing. To that end, CADRe is leading a Delphi study to establish minimum components of informed consent for genetic testing [39]. CADRe plans to seek input from additional stakeholders, such as individuals who have undergone genetic testing, electronic health records companies, and genetic testing laboratories, to integrate this framework at the point of care. Given some evidence that primary care providers lack knowledge, skill, and confidence to discuss genetic testing [40, 41], this additional support for primary care and other clinicians will be critical to the safe and effective implementation of the framework. Including experts, patients, and other stakeholders in development of this support will help bring to fruition the CADRe assumption that clinicians will identify genetic risk, choose testing, and communicate results appropriately.

It is important to acknowledge the limits in scope of the CADRe framework presented here. This study focuses mainly on adult-onset indications (hereditary cancer, cardiovascular, and neurological) and pediatric testing in the context of the ACMG SF v2.0 genes [24] and exome testing, and does not address some common genetic testing situations, for example indications related to prenatal genetic testing and other pediatric genetic testing scenarios. Furthermore, there are clinical situations not included in the CADRe rubric in which a traditional GC approach may be appropriate. GC has been shown to provide benefits such as empowerment and self-efficacy even in the absence of the availability of genetic testing, as in a psychiatric GC clinic [42]. Genetic counselors may additionally find their expertise called upon in areas such as precision medicine testing, where patients (or genetic testing consumers) may initiate GC to discuss their genomic results that modify their chances of developing a multifactorial condition such as heart disease [43].

One potential limitation of our approach to using the CADRe rubrics is the method of small subgroups of workgroup members making the initial recommendation determinations. However, we anticipate the process of developing consensus, checking for consistency across condition-indication pairs, and resolving discrepancies as a full working group for particularly challenging indication-condition pairs mitigated potential biases of the sub-groups. Finally, while the CADRe rubrics were developed by individuals with expertise in genomics and iteratively updated based on feedback from focus groups and interviews with genetics and non-genetics clinicians as well as patient stakeholders, the rubrics and communication recommendations have yet to be tested in practice. Further input from stakeholders on targeted discussion and brief communication approaches and studies comparing the effectiveness of CADRe-informed care versus usual care will be critical next steps for determining the utility of this communication framework in providing quality patient care and experience, as well as improved access to genetic information across healthcare.

## Conclusions

The CADRe recommendations support pre-test discussions moving towards targeted discussions for many tests and indications, which could lead to shorter appointment times and a shift towards non-genetics clinicians completing pre-test genetic risk assessment and genetic testing at the point-of-care. Longer traditional GC consultations would be prioritized to patients with more complex and uncertain pre- and post-test discussions where genetics expertise can best support families needing detailed information, education, and psychological support. The CADRe recommendations provide needed guidance for clinicians in determining the depth of pre- and post-test communication, thus facilitating the wider integration of genetic testing in healthcare.

## Supplementary Information

**Additional file 1: Supplementary Methods**. Detailed description of methods for operationalizing the CADRe pre- and post-test rubrics.

**Additional file 2: Supplementary Table S1**. All CADRe communication recommendations for the standard testing indications.

**Additional file 3: Supplementary Table S2**. CADRe communication recommendations for unique testing indications.

## Data Availability

All data generated or analyzed during this study are included in this published article and its supplementary information files.

## Abbreviations

ACMG SF: American College of Medical Genetics secondary findings
B/LB: Benign/likely benign/no variant
CADRe: Consent and Disclosure Recommendations working group
FH: Familial hypercholesterolemia
GC: Genetic Counselor
MAP: *MUTYH*-associated polyposis
MHS: Malignant hyperthermia susceptibility
P/LP: Pathogenic/likely pathogenic variants
