# Supplementary material for: Application of a framework to guide genetic testing communication across clinical indications
Source: Genome Med. 2021 Apr 29;13:71. doi: 10.1186/s13073-021-00887-x (PMC8086064; doi:10.1186/s13073-021-00887-x)
Supplement: Supplementary file 1 — Additional file 1: Supplementary Methods. Detailed description of methods for operationalizing the CADRe pre- and post-test rubrics. [file 13073_2021_887_MOESM1_ESM.docx]

**Supplementary Methods**

Operationalizing the Framework as Online Surveys

Each element of the CADRe rubric was presented in the online survey for each condition-indication pair, operationalized as follows:

*Testing Complexity*: testing complexity was defined as increased when at least two of the following criteria were met: increased risk of incidental findings, increased risk of a VUS, lower clinical validity (e.g., lower sensitivity/specificity) of the testing, or poorly understood genetic etiology of a condition. Given our approach of using the framework in a condition-by-condition manner, the consensus of the WG was to define all the ACMG SF v2.0 conditions,^26^ as well as the cancer and cardiovascular moderate risk and , as “less complex” in terms of testing. This category was included in the surveys for the neurodegenerative risk genes.

*Risk of Adverse Psychological Impact:* defined as published evidence that genetic testing for the particular condition is associated with increased risk of adverse psychological outcomes (e.g., depression, anxiety, distress, coping concerns, or suicidal ideations). This risk can be, for example, related to psychological responses to the detection of pathogenic/likely pathogenic variants, or negative results leading to survivor guilt. A theoretical concern about adverse psychological outcomes of a test without supporting evidence was insufficient to meet this criterion. This element was operationalized by completing a preliminary literature search in PubMed using the name of the condition or the specific gene name and any of the following terms: psychological, depression, anxiety, distress, coping, suicide, or survivor guilt. Pertinent articles were included in the rubrics for working group members to review during the rubric completion process. Additionally, working group members were encouraged to complete their own literature search to identify any additional concerns of adverse psychological impact.

*Significant potential for sudden death:* defined as a condition associated with a non-minimal risk of sudden death (e.g., dilated cardiomyopathy, Marfan syndrome). This was operationalized as having a ClinGen Actionability WG score of a severity three^25^. For genes not curated by the Actionability WG we asked respondents to provide any evidence a condition had significant potential for sudden death.

*Management of condition clinically complex:* defined as identification of a pathogenic/likely pathogenic variant would lead to: (1) the need for a discussion between the patient (or family) and clinician to develop a detailed or complex clinical plan; (2) interventions that carry significant patient burden (e.g., longitudinal multidisciplinary care, significant impact on daily functioning (e.g. dietary restrictions or daily medication requiring monitoring); OR (3) interventions that involve substantial morbidity/mortality risk (e.g. surgical interventions). This was operationalized using data from the ClinGen Actionability Workgroup that quantified the burden of interventions associated with specific gene-condition pairs. If the Actionability Workgroup scored a gene-intervention pair for managing disease risk as being particularly burdensome (i.e., score of 0 or 1), we considered the management of the condition clinically complex. If there were multiple interventions associated with any given gene-condition pairing, the most burdensome gene-intervention score was used in our rubric^25^.

*Educational materials*: defined as material(s) written in lay language, broadly accessible, culturally appropriate, and sufficiently covering the relevant consent or disclosure issues. This was operationalized by conducting a preliminary search for patient friendly educational materials which were included in the surveys for working group members to review. Search terms included the name of the condition and any combination of: “support group”, “education”, and “resources”. Group members were encouraged to search for additional educational materials.

*Significant residual risk for disease given negative genetic test results:* Testing was considered to have a significant residual risk for disease when there was limited clinical validity/sensitivity for the gene-condition pairing, or when there was a risk for phenotype based on family history. If the indication for testing was suggestive family history, this was considered yes. For all other indications, risk for phenotype based on family history was considered to be an individual patient level factor and unable to be determined for the rubric.

Exome sequencing was classified by providing the subgroup members with an indication for testing. Subgroup members used the principles of the framework to determine whether the level of communication would be consistent with the overall framework.
